# Chronic antibiotic use during adulthood and weight change in the Sister Study

**DOI:** 10.1371/journal.pone.0216959

**Published:** 2019-05-16

**Authors:** Melissa Furlong, Sandra Deming-Halverson, Dale P. Sandler

**Affiliations:** 1 Department of Epidemiology, University of North Carolina at Chapel Hill, Chapel Hill, NC, United States of America; 2 Department of Community, Environment, and Policy, University of Arizona, Tucson, AZ, United States of America; 3 Social & Scientific Systems, Inc., Durham, NC, United States of America; 4 Division of Epidemiology, Vanderbilt University Medical Center, Nashville, TN, United States of America; 5 Epidemiology Branch, National Institute of Environmental Health Sciences, Durham, NC, United States of America; McMaster University, CANADA

## Abstract

**Background/Objectives:**

Antibiotic use in early life has been associated with weight gain in several populations. However, associations between chronic antibiotic use and weight among adults in the general population are unknown.

**Subjects/Methods:**

The NIEHS Sister Study is a longitudinal cohort of sisters of women with breast cancer. We examined associations between chronic antibiotic use (≥ 3 months) during the fourth decade of life (30–39 years) and subsequent obesity at enrollment (mean age = 55) via logistic regression. We also examined associations between chronic antibiotic use in the 5 years and 12 months prior to enrollment and weight gain after enrollment in linear mixed models. Models were adjusted for race/ethnicity, education, urban/rural status, age, and smoking.

**Results:**

In adjusted analyses (n = 50,237), chronic penicillin use during the 4^th^ decade of life was associated with obesity at enrollment (OR 2.00, 95% CI 1.40, 2.87), and use in the 5 years prior to enrollment was associated with increased BMI change after enrollment (β 1.00 95% CI 0.01, 2.00). Use of bactericidals (OR 1.71, 95% CI 1.29, 2.26) during the 4^th^ decade of life was also associated with obesity at enrollment. Associations for penicillins and bactericidals were consistent across indications for use. Bacteriostatic use in the 5 years prior to enrollment was associated with a reduction in BMI after enrollment (β -0.52, 95% CI -1.04, 0.00), and tetracycline use during the 4^th^ decade of life was associated with reduced odds of obesity at enrollment (OR 0.72, 95% CI 0.56, 0.92). However, these inverse associations were only present for those who reported taking antibiotics for skin purposes. Cephalosporins, macrolides, quinolones, and sulfonamides were not associated with BMI change over time.

**Conclusions:**

Chronic use of antibiotics during adulthood may have long-lasting impacts on BMI. Associations may differ by antibiotic class, and confounding by indication may be important for some antibiotic classes.

## Introduction

Antibiotics have been used for growth promotion in several species of livestock since the 1950s [1, 2], although the hypothesis that antibiotic use may similarly result in weight gain in healthy humans has only recently been investigated. Oral antibiotics increase height and weight of children with malnutrition or infection in low and middle income countries (reviewed in [3]). Antibiotic treatment of severe, chronic diseases in adults has also been associated with weight gain. Macrolide treatment for cystic fibrosis [4]; omeprazole, amoxicillin, and clarithromycin combination treatment for *Helicobacter pylori* [5]; and treatment of infective endocarditis with vancomycin and gentamycin [6]; were all associated with increased weight gain in treated patients. These weight gain effects may have been mediated by the treatment of infectious disease, since infections may lead to reduced absorption of nutrients, increased loss of nutrients from diarrhea, nutrient diversion from growth to immune support, and lack of appetite [7–9].

Other evidence supports a microbiome-mediated pathway for the relationship between antibiotics and obesity. Chronic and repeated antibiotic and probiotic use may have a profound impact on the composition of the gut microbiome [10–12], which differs between obese and lean people [13–15]. In animal and human studies, some probiotic strains have been associated with weight gain (reviewed in [16]), though others have shown associations with weight loss [17–19]. Since probiotics do not treat infections, at least some of the mechanism by which probiotics cause weight gain may be via alteration of gut microbiota and not via a protective effect against harmful pathogens. Transplanting fecal microbiota from lean/obese discordant human twin pairs into germfree mice led to weight gain in the mice that received the microbiota from the obese twins [20], implying that the microbiota themselves may be responsible for weight alteration.

Bacteria may influence the host’s weight via several mechanisms. Bacteria are responsible for the metabolism of complex carbohydrates that result in short chain fatty acids [21], which provide an additional source of energy for the body through lipid and glucose synthesis and absorption of monosaccharides from the gut lumen [22]. This absorption induces hepatic lipogenesis, is responsible for depositing triglycerides in fat cells, and generally influences energy harvest from the diet and storage of energy in the host [23].

In humans, there has been a recent explosion of epidemiological evidence supporting an association between early-life antibiotics and overweight and obesity in children [24–33], although indication for use may be an important confounder [32]. As the first year of life is critical for development of the microbiota, this research suggests that disruption of the development of the microbiome during infancy may lead to increased BMI in later life, and the effect may vary by type of antibiotic. However, because microbiota are responsible for energy harvesting and storage throughout life, and because even week-long doses of antibiotics may alter microbial composition for 2 years in adults [34], chronic antibiotic exposure in adulthood may also result in altered weight. This has not been previously explored from a chronic disease perspective, in part because long-term antibiotic use is somewhat rare. To address this gap, we conducted an analysis to examine the association of different antibiotics with weight and weight change in a large prospective cohort of adult women.

## Methods

### Study population

The National Institute of Environmental Health Sciences Sister Study is a prospective cohort study of environmental and genetic risk factors for breast cancer and other endpoints in approximately 50,000 women ages 35 to 74 years [35]. At enrollment, participants were unaffected with breast cancer but did have a sister who was diagnosed with the disease. Women were recruited from all 50 states in the USA and Puerto Rico. Although they are similar to the general population with regard to some lifestyle factors, as with most volunteer cohorts, participants tend to be healthier and to have higher socioeconomic status than similarly aged women from the general population. We used data from Sister Study Data Release 4.1, which contains baseline data and two detailed follow-ups that include height and weight. Details of the study have been described elsewhere. For this study, all women who were not currently pregnant or breastfeeding with complete data on anthropometry at enrollment and anthropometric history, history of antibiotic usage, and covariates were included in analyses. This research was approved by the Institutional Review Boards of the National Institute of Environmental Health Sciences, NIH, and the Copernicus Group.

### Anthropometric measurement

At enrollment (ages 35–74, mean age = 55), examiners visited participant’s homes and measured height and weight. On the enrollment questionnaire, women reported height and weight during their 30’s. Additionally, at the first and second detailed follow-ups, women self-reported current height and weight. Body mass index (BMI) was calculated using the formula weight (lb)/ [height(in)]^2^ x703, and we used the CDC adult conventions to determine weight status [36]. BMI below 18.5 is considered underweight, 18.5 to <25.0 is normal weight, 25.0 to <30 is overweight, and 30.0 and above is obese.

Percent changes in BMI were also calculated for changes from a woman’s 30s (4^th^ decade of life) to enrollment, from enrollment to the first detailed follow-up, from enrollment to the second detailed follow-up, and from the first to the second detailed follow-up. At enrollment, we used BMI from the examiner-measured height and weight data, and at all other instances used the self-report. In sensitivity analyses, we evaluated self-reported BMI at enrollment.

### Antibiotic use

At enrollment, women reported whether they had ever taken oral antibiotics at least three times a week for three months or longer, excluding topical antibiotics. They additionally reported the name of the antibiotic, the indication for use, the age they first started taking the antibiotic regularly, and the duration of use.

We collapsed antibiotics into classes for analyses, including aminoglycosides, cephalosporins, chloramphenicol, macrolides, penicillins, other beta lactams, quinolones, sulfonamides, tetracyclines, and other antibiotics. We also grouped antibiotics by whether they are bactericidal (penicillins, quinolones, cephalosporins, aminoglycosides, other beta-lactams) or bacteriostatic (tetracyclines, macrolides, sulfonamides, chloramphenicol). We collapsed indications into categories of skin issues (i.e. rosacea, acne), and non-skin issues (ear nose and throat, respiratory, prevention [taking prior to surgery or dental visits], sinus, urinary tract infections, other), since this categorization was the best predictor of weight change. In analyses of individual antibiotics, we considered binary variables indicating ever-use of antibiotic classes with at least 20 exposed participants, thus, we excluded aminoglycosides (n = 13), chloramphenicols (n = 5), and other beta lactams (n = 2). We also considered antibiotic use during a woman’s 30s, antibiotic use in the year prior to enrollment, and antibiotic use in the five years preceding enrollment.

### Covariates

To identify important covariates for estimating the relationship between chronic antibiotic use and weight gain, we considered a directed acyclic graph (DAG) to identify possible confounders, colliders, and mediators [37]. Variables in the DAG included race/ethnicity, education, age at enrollment, urban/rural status, smoking, exercise, diabetes, sugary beverage intake at enrollment, total calorie intake at enrollment, and healthy lifestyle. All models were adjusted for race/ethnicity (non-Hispanic white, non-Hispanic black, Hispanic, other), education (binary variable indicating at least bachelor’s degree), age at enrollment (four categories included <45, 45 to <55, 55 to <65, and over 65), urban/rural status (binary variable indicating urban residence at enrollment), and smoking status (total pack years, calculated from packs/day * years smoked). Distributions of these variables were calculated for the populations at enrollment and at each follow-up. We also calculated the length of time participants reported using each antibiotic class, and report the medians and the 25^th^ and 75^th^ percentiles for each class. These measures are included only for those who report using antibiotics.

### Data analysis

We estimated prevalence odds ratios of the associations between ever use of antibiotics and obesity at enrollment in logistic regression models. We also estimated associations between use of antibiotics during the 30s and obesity at enrollment (minimum age at enrollment = 35, mean age = 55). To assess this, we considered those who initiated antibiotic use during their 30s as exposed. Those who initiated antibiotic use outside of that timeframe were excluded. This approach addressed the concern that initiation of antibiotic use prior to their 30s might capture weight gain prior to their 30s. It also addressed the concern that initiation of antibiotic use after the 4^th^ decade of life might not represent a model where the exposure precedes weight gain, and also narrows the time window between last reported weight and initiation of antibiotic use. We also estimated associations between use of antibiotics during their 30s and obesity at enrollment in a logistic regression model that excluded women with obesity during their 30s.

We also took advantage of more recently collected data to minimize recall bias of weight and antibiotic use. We estimated associations between reported antibiotic use in the 12 months prior to enrollment, and five years prior to enrollment, and percent changes in BMI from enrollment to each follow up and from the 1^st^ to the 2^nd^ follow up. We used a linear mixed regression model with random effects for subject, fixed effects for the covariates listed previously, and unstructured covariance to perform longitudinal analyses. In addition to controlling for the standard set of covariates, we controlled for BMI at enrollment. In sensitivity analyses, we omitted this variable. Since several participants reported using multiple antibiotic classes, and since users of other antibiotics were included in the referent categories for several models, we also performed sensitivity analyses where the exposed category included only those who reported using the antibiotic class of interest, and the referent category only included those who reported no antibiotic use. Participants who reported using other antibiotic classes were excluded from these models.

To account for possible confounding by indication, we separately considered associations between antibiotics taken for skin and antibiotics taken for non-skin purposes in modeling antibiotic associations with weight gain. Women who did not use antibiotics were not asked about these conditions or, if asked, asked in the same way as women who used antibiotics, and therefore we were unable to assess interactions between antibiotics and indications. Instead, we compared associations for antibiotic users who reported uses for skin, and users who reported uses for non-skin, and compared those against participants who did not report using that antibiotic.

We conducted several sensitivity analyses. These included evaluating modification by race/ethnicity, and diabetes status, at an alpha of 0.10. We controlled for time between first and second follow ups in longitudinal models, and also separately evaluated whether associations between antibiotic use differed by age at initiation. To assess this, we examined childhood initiation (<15 years), early adult initiation (15–45), and menopause initiation (45–55), and associations with obesity at enrollment, restricted to participants who were over the age of 55.

All statistical analyses were performed in R V3.3.1.

## Results

Women in the Sister Study were predominantly white, between 40 and 60 years of age at enrollment, of normal weight, and approximately half had a college degree (Table 1). Of 50,884 women who were enrolled, 50,237 had complete covariate, exposure, and weight data and met inclusion criteria. Of the included women, 46,697 (93%) completed the first follow-up and 44,381 (88%) completed the second follow-up. Demographic composition was stable over follow-up (Table 1). The first and second follow-ups took place on average 2.8 (sd = 0.6) and 5.7 (sd = 1.0 years) years after enrollment, respectively. The median length of use was 12 months for macrolides, penicillins, and sulfonamides, 6 months for cephalosporins and quinolones, and 24 months for tetracyclines (Table 1).

**Table 1:** Characteristics of study population by follow-up.

Characteristic		Total Population at Enrollment	1^st^ Follow-up	2^nd^ Follow-up
		N = 50,237	N = 46,697	N = 44,381
Continuous Variables (Mean (SD))				
	BMI in 30s	23.30 (3.99)	23.24 (3.94)	23.19 (3.84)
	Smoking in pack years	6.40 (12.48)	6.28 (12.33)	6.22 (12.21)
	Current Smoking (Any) n (%)	4,056 (8.07)	3,565 (7.63)	3,285 (7.40)
Categorical Variables (n (%))				
Education	No College	24,615 (49.00)	22,539 (48.27)	21,103 (47.54)
	College Degree	25,622 (51.00)	24,158 (51.73)	23,278 (52.46)
Race/ Ethnicity	NH white	42,073 (83.75)	39,579 (84.76)	38,436 (86.60)
	NH Black	4,345 (8.71)	3,779 (8.09)	3,070 (6.91)
	Hispanic	2,477 (4.93)	2,149 (4.60)	1,738 (3.92)
	Other	1,313 (2.61)	1,190 (2.55)	1,137 (2.56)
Urban	Rural	21,099 (41.98)	19,671 (42.12)	18,753 (42.25)
	Urban	29,162 (58.02)	27,026 (57.88)	25,628 (57.75)
Age at Enrollment	35–44	6,461 (12.87)	5,856 (12.54)	5,478 (12.34)
	45–54	17,330 (34.50)	15,990 (34.24)	15,216 (34.28)
	55–64	17,712 (35.26)	16,672 (35.70)	15,923 (35.88)
	≥ 65	8,734 (17.39)	8,179 (17.51)	7,764 (17.49)
Ever Chronic Antibiotic Use	None	44,977 (89.53)	41,785 (89.48)	39,694 (89.37)
	Any	5,260 (10.47)	4,912 (10.52)	4,720 (10.63)
Length of Antibiotic Use* (Median, 25/75 percentile in months)	Cephalosporins	6 (3, 12)	6 (3, 12)	6 (3, 12)
	Macrolides	12 (6, 30)	12 (6, 24)	12 (6, 24)
	Penicillins	12 (4, 36)	12 (4, 36)	12 (4, 36)
	Quinolones	6 (3, 12)	6 (3, 12)	6 (3, 12)
	Sulfonamides	12 (6, 36)	12 (6, 36)	12 (6, 36)
	Tetracyclines	24 (12, 36)	24 (12, 36)	24 (12, 36)

Total population at enrollment includes participants at enrollment who met inclusion criteria for the current study, 1^st^ follow-up includes participants who returned the 1st follow-up questionnaire and had BMI information, 2^nd^ follow-up includes participants who returned the 2nd follow-up questionnaire and had BMI data.

*Medians and 25/75 percentiles for antibiotic use are calculated based only on those who reported ever using that specific class

In adjusted analyses of the associations between ever-use of antibiotics and obesity at enrollment, ever-use of cephalosporins (OR 1.78, 95% CI 1.38, 2.29), penicillins (OR 1.35, 95% CI 1.20, 1.52), quinolones (OR 1.69, 95% CI 1.37, 2.09), and bactericidal antibiotics (OR 1.43, 95% CI 1.29, 1.58) were associated with an increased odds of obesity at enrollment (Table 2). Ever-use of tetracyclines (OR 0.76, 95% CI 0.70, 0.83) and bacteriostatic antibiotics (OR 0.84, 95% CI 0.78, 0.91) were associated with a lower odds of obesity (Table 2). Macrolides and sulfonamides showed no association between ever-use and obesity at enrollment. Similarly, ever use of any antibiotic was not associated with obesity at enrollment. Similar associations were evident at the 1^st^ and 2^nd^ follow-ups, and for both adjusted and unadjusted ORs.

**Table 2:** Prevalence odds ratios of associations between antibiotic use and obesity.

			At Enrollment N = 50,237		At 1^st^ Follow-up N = 46,697		At 2^nd^ Follow-up N = 44,381	
	*Not Obese	Obese	Unadjusted OR (95% CI)	Adjusted OR (95% CI)^1^	Unadjusted OR (95% CI)	Adjusted OR (95% CI)^1^	Unadjusted OR (95% CI)	Adjusted OR (95% CI)^1^
No Cephalosporins	35,114	14,872	**1.78 (1.39, 2.29)**	**1.78 (1.38, 2.29)**	**1.54 (1.18, 2.01)**	**1.53 (1.17, 2.00)**	**1.57 (1.20, 2.07)**	**1.58 (1.20, 2.08)**
Ever Cephalosporin	143	108						
No Macrolides	34,780	14,788	0.95 (0.80, 1.12)	1.03 (0.87, 1.22)	1.08 (0.91, 1.28)	1.15 (0.97, 1.37)	1.10 (0.92, 1.30)	1.15 (0.97, 1.37)
Ever Macrolides	477	192						
No Penicillins	34,479	14,512	**1.43 (1.27, 1.61)**	**1.35 (1.20, 1.52)**	**1.35 (1.19, 1.53)**	**1.27 (1.12, 1.44)**	**1.29 (1.14, 1.47)**	**1.21 (1.06, 1.38)**
Ever Penicillins	778	468						
No Quinolones	35,050	14,825	**1.77 (1.44, 2.18)**	**1.69 (1.37, 2.09)**	**1.50 (1.19, 1.88)**	**1.44 (1.14, 1.81)**	1.27 (0.99, 1.62)	1.21 (0.94, 1.55)
Ever Quinolones	207	155						
No Sulfonamides	34,915	14,833	1.01 (0.83, 1.23)	1.03 (0.84, 1.25)	1.07 (0.88, 1.31)	1.09 (0.89, 1.34)	1.14 (0.93, 1.39)	1.16 (0.94, 1.42)
Any Sulfonamides	342	147						
No Tetracyclines	32,848	14,262	**0.69 (0.63, 0.75)**	**0.76 (0.70, 0.83)**	**0.71 (0.65, 0.77)**	**0.77 (0.71, 0.85)**	**0.75 (0.68, 0.82)**	**0.81 (0.74, 0.89)**
Ever Tetracyclines	2,409	718						
No Antibiotics	31,494	13,483	0.93 (0.87, 0.99)	0.98 (0.92, 1.05)	0.92 (0.86, 0.98)	0.96 (0.89, 1.02)	0.94 (0.88, 1.00)	0.97 (0.91, 1.04)
Ever Any Antibiotic	3,763	1,497						
No Bactericidals	34,207	14,007	**1.50 (1.36, 1.65)**	**1.43 (1.29, 1.58)**	**1.39 (1.25, 1.54)**	**1.32 (1.18, 1.47)**	**1.33 (1.19, 1.49)**	**1.26 (1.13, 1.41)**
Ever Bactericidal	1,050	658						
No Bacteriostatics	32,323	14,007	**0.77 (0.71, 0.83)**	**0.84 (0.78, 0.91)**	**0.79 (0.73, 0.85)**	**0.85 (0.79, 0.92)**	**0.83 (0.77, 0.90)**	**0.89 (0.82, 0.96)**
Ever Bacteriostatics	2,934	973						

*Obese case numbers reflect obesity status at enrollment. N’s for each time point reflect those with complete covariate and outcome data.

^1^Covariates for adjusted models include race/ethnicity, education, urban/rural, age, and total smoking pack years

Associations between antibiotic use during the 4^th^ decade of life (30s) and obesity at enrollment resulted in slightly different findings. Use of penicillins (OR 2.00, 95% CI 1.40, 2.87) and bactericidals (OR 1.71, 95% CI 1.29, 2.26), but not cephalosporins, quinolones or the broad “any antibiotic” category, during their 30s was associated with obesity at enrollment (Table 3). Initiating use of tetracyclines (OR 0.72, 95% CI 0.56, 0.92), or any bacteriostatic antibiotic (OR 0.82, 95% CI 0.67, 1.01), during a woman’s 30s was inversely associated with obesity (Table 3). The magnitudes of the associations for penicillins and bactericidals were similar across categories of indications for use (Table 4). The OR for skin was non-significant for penicillins and bactericidals, but few participants reported use of these antibiotics for skin conditions (n = 21 for penicillins, n = 30 for bactericidals). The inverse association between tetracyclines and obesity was only present for skin-users (OR 0.64, 95% CI 0.48, 0.85); the OR for non-skin condition use was 1.19, 95% 0.70, 2.02. Similarly, the inverse association between bacteriostatic use and obesity was only seen for skin users.

**Table 3:** Incidence odds ratios of associations for initiating antibiotic use during the 4^th^ decade of life with obesity at enrollment.

	^1^Not Obese	Obese	Adjusted OR (95% CI)^2^
No Cephalosporins^1^	34,659	11,898	1.52 (0.90, 2.55)
Ever Cephalosporin	44	22	
No Macrolides	34,326	11,832	1.01 (0.65, 1.55)
Ever Macrolides	93	28	
No Penicillins	34,044	11,620	**2.00 (1.40, 2.87)**
Ever Penicillins	77	52	
No Quinolones	34,595	11,853	1.02 (0.50, 2.09)
Ever Quinolones	34	10	
No Sulfonamides	34,462	11,853	1.11 (0.68, 1.81)
Any Sulfonamides	63	22	
No Tetracyclines	32,411	11,412	**0.72 (0.56, 0.92)**
Ever Tetracyclines	342	77	
No Antibiotics	31,269	10,851	1.01 (0.85, 1.19)
Ever Any Antibiotic	594	189	
No Bactericidals	33,775	11,464	1.71 (1.29, 2.26)
Ever Bactericidal	144	80	
No Bacteriostatics	31,891	11,197	0.82 (0.67, 1.01)
Ever Bacteriostatics	470	120	

^1^Use reflects initiation of chronic antibiotic use during the 4^th^ decade of life (30s). Models exclude women who reported being obese during their 30’s

^2^All models adjusted for race/ethnicity, education, urban/rural, age, and total smoking pack years.

**Table 4:** Associations between antibiotic use^1^ during the 4^th^ decade of life and obesity at enrollment, according to indications for use^3^.

		N Not obese/Obese	OR^2^ (95% CI)
Macrolides			
	No Macrolide Use	34,326 / 11,832	Ref
	Macrolides for Skin	56/15	0.97 (0.54, 2.1.73)
	Macrolides for Non-Skin	37/13	1.06 (0.56, 2.01)
Penicillins			
	No Penicillin Use	34,044 / 11,620	Ref
	Penicillins for Skin	13/8	2.32 (0.94, 5.73)
	Penicillins for Non-Skin	64/44	**1.95 (1.32, 2.88)**
Sulfonamides			
	No Sulfonamide Use	34,462/ 11,853	Ref
	Sulfonamides for Skin	16/3	0.69 (0.20, 2.39)
	Sulfonamides for Non-Skin	47/19	1.23 (0.72, 2.11)
Tetracyclines			
	No Tetracycline Use	32,411 / 11,412	Ref
	Tetracyclines for Skin	290/58	0.64 (0.48, 0.85)
	Tetracyclines for Non-Skin	52/19	1.19 (0.70, 2.02)
Bactericidals			
	No Bactericidal Use	33,775/ 11,464	Ref
	Bactericidals for Skin	19/11	**2.16 (1.01, 4.61)**
	Bactericidals for Non-Skin	125/69	**1.65 (1.22, 2.23)**
Bacteriostatics			
	No Bacteriostatic Use	31,891 / 11,197	Ref
	Bacteriostatics for Skin	342/73	**0.70 (0.54, 0.90)**
	Bacteriostatics for Non-Skin	128/47	1.12 (0.0.80, 1.57)

^1^Use reflects initiation of chronic antibiotic use during the 4^th^ decade of life (30s). Models exclude women who reported being obese during their 30’s

^2^All models adjusted for race/ethnicity, education, urban/rural, age, and total smoking pack years

^3^Cephalosporins and quinolones had fewer than 10 reported users for skin and are excluded

Findings for penicillin were similar when we examined associations between reported antibiotic use immediately prior to enrollment and weight gain during the follow-up periods (Table 5). Penicillin use in the 5 years prior to enrollment was associated with increased weight gain (% change in BMI) after follow-up (β 1.00, 95% CI 0.01, 2.00). Although the beta estimate for penicillin use in the 12 months prior to enrollment was similarly elevated, the association was not significant (β 1.53, 95% CI -025, 3.31). These effect sizes are relatively modest; using penicillin in the past 5 years was associated with a 1% gain in BMI. For example, for a 5’5” woman who weighed 150 pounds at enrollment, this represents a gain of approximately 1.5–2 pounds over the entire follow-up period. Conversely, bacteriostatic use in the 5 years prior to enrollment was associated with weight loss over follow-up (β -0.52, 95% CI -1.04, 0.00), although there was no significant association for bacteriostatic use 12 months prior to enrollment (β 0.28, 95% CI -0.67, 1.22). In contrast to results from models examining use in earlier life (initiation of use in 30s), examining use in the 12 months or 5 years prior to enrollment resulted in no consistent association between tetracyclines or bactericidals and weight change after enrollment. For all models, associations were unchanged in sensitivity analyses where the exposed category included only those who reported using the antibiotic of interest, and the referent category included only those who reported never taking any antibiotics.

**Table 5:** Mixed models of longitudinal associations between antibiotic use before enrollment and percentage change in body mass index between follow-ups.

	Antibiotic use 12 months Prior To Enrollment β (95% CI)	Antibiotic Use 5 years Prior To Enrollment β (95% CI)
Cephalosporins N Use/No Use	26/48,099	72/48,053
Cephalosporins β (95% CI)	0.06 (-2.31, 2.42)	-0.21 (-1.63, 1.20)
Macrolides N Use/No Use	25/48,080	99/48,006
Macrolides β (95% CI)	-0.55 (-2.94, 1.84)	-0.92 (-2.12, 0.28)
Penicillins N Use/No Use	48/48,046	152/47,942
Penicillins β (95% CI)	1.53 (-0.25, 3.31)	**1.00 (0.01, 2.00)**
Quinolones N Use/No Use	33/48,087	165/47,955
Quinolones β (95% CI)	-1.18 (-3.29, 0.94)	-0.52 (-1.46, 0.42)
Sulfonamides N Use/No Use	19/48,095	83/48,031
Sulfonamides β (95% CI)	-1.61 (-4.50, 1.27)	-0.60 (-1.92, 0.72)
Tetracyclines N Use/No Use	121/47,970	271/47,720
Tetracyclines β (95% CI)	0.80 (-0.29, 1.90)	-0.43 (-1.05, 0.20)
Bactericidals N Use/No Use	103/48,031	366/47,768
Bactericidals β (95% CI)	0.71 (-0.50, 1.91)	0.04 (-0.60, 0.67)
Bacteriostatics N Use/No Use	163/47,971	534/47,600
Bacteriostatics β (95% CI)	0.28 (-0.67, 1.22)	-0.52 (-1.04, 0.00)

Linear Regression with random effects for subject and visit. BMI reported at enrollment, at first follow-up, and second follow-up. Percent change in BMI calculated for time between enrollment and first follow-up, time between first follow-up and second follow-up, and time between enrollment and second follow-up. All models adjusted for race/ethnicity, education, urban/rural, age, smoking, and BMI at enrollment. Results are similar without controlling for BMI at enrollment.

Sensitivity analyses included assessing modification by race/ethnicity and diabetes status, confounding by time between first second follow-ups, and age at antibiotic initiation. None of these analyses influenced associations or changed interpretations.

## Discussion

Across all analyses, penicillin use was prospectively associated with weight gain or obesity, which appeared independent of indication for use. Although bactericidal use during the 4^th^ decade of life was associated with obesity at enrollment, the association between bactericidal use immediately prior to enrollment and subsequent weight gain was not significant. Although use of tetracyclines and bacteriostatics were associated with reduced odds of obesity among those taking antibiotics for skin purposes, there was no association for participants taking these antibiotics for non-skin purposes. Additionally, most bacteriostatics users were tetracyclines users, and this association was likely driven by tetracyclines. Finally, ever-use of cephalosporins, quinolones, penicillins and bactericidals were cross-sectionally associated with obesity at enrollment, although cephalosporins and quinolones were not associated with weight gain in prospective analyses that minimized the possibility of reverse causality. Reverse causality is not a trivial concern, since the obese population has a higher risk of infection than the non-obese population [38].

The association between antibiotics and weight change is biologically plausible and potentially mediated by changes in the gut microbiota and alterations in hormones that regulate energy homeostasis, such as leptin and ghrelin [23]. Antibiotic exposure does alter the composition of microbial communities (reviewed in [34]). However, there is a wide range of microbial action across antibiotic classes and antibiotic targets. Tetracyclines and bacteriostatics were paradoxically associated with reduced weight, although only among those using tetracyclines for skin conditions. This suggests the possibility of confounding by indication for these classes of antibiotics. Although associations between penicillins and bactericidals were consistent across indications, the number of participants who reported using these antibiotics for skin conditions was relatively small. Prior research has also suggested that infections, and not antibiotics, drive obesity [32]. Although this possibility cannot be ruled out in our study, if this were true we would expect to see an overall effect for antibiotic use and obesity in our population, across antibiotic classes. However, we only report a consistent association for penicillins, which argues against this potential confounder. Alternatively, if penicillins tend to be prescribed more after intestinal surgery in response to longer and more resistant infections, compared to other antibiotics, this may be another possible confounding factor. Possible explanation for the differences may be due to differences in targeted action; for instance, later generations of penicillins are generally considered to be broad-spectrum antibiotics. Researchers have speculated that broad-spectrum and narrow-spectrum antibiotics may differentially alter bacterial diversity in humans [39]. Interestingly, tetracyclines are also considered broad-spectrum antibiotics, and these classes generally had opposite effects on obesity. It is plausible that broad-spectrum antibiotics may be more likely to influence weight changes through broad alterations in the microbiome, and the direction of this weight change (up or down) may be a function of the specific antibiotic class.

Differing results from the cross-sectional analyses and longitudinal analyses could be due to a few factors. First, people who are overweight or obese are more likely to acquire an infection [38] and thus need antibiotics. These individuals may also be less likely to engage in physical activity due to their health status. Any cross-sectional analysis of a population that reports associations between antibiotics and obesity will capture some of this phenomenon. Second, the populations in the cross-sectional and longitudinal analyses are different. The cross-sectional analyses classify everyone who ever chronically used an antibiotic as exposed, while the longitudinal analyses classifies only those who used antibiotics in their 30s, or those who used antibiotics in the 12 or 60 months prior to enrolling in the Sister Study, as exposed. Antibiotic use at different time points in life may have varying effects–puberty, pregnancy, and menopause are all hormonally driven events that might modify any effects of antibiotics on weight gain. These time periods, with the exception of pregnancy, were mostly excluded from the prospective analyses, but included in cross-sectional analyses. Additionally, analyses that predict weight gain after enrollment based on pre-enrollment antibiotic use will fail to capture many of those who experienced weight gain immediately after antibiotic use, biasing those results towards the null. These effects would, however, be captured in the cross-sectional analyses.

In general, previous studies of antibiotic use during infancy and overweight and obesity in children’s cohorts have implicated exposure to multiple classes of antibiotics rather than penicillins alone [26, 27, 33]. However, one study reported adverse associations with penicillins, cephalosporins, and macrolides [25], and a randomized clinical study in adults reported weight gain in patients treated for *H*. *pylori* with amoxicillin [5]. Another cohort reported no effect for penicillins (a narrow spectrum antibiotic) but did report effects for broad-spectrum antibiotics [27]. Infancy presents a very different microbial developmental window, and different antibiotics may have differing impacts on the microbiota of infants relative to adults. Additionally, others included short-term use of antibiotics while we examined longer-term, chronic use. Differences in effects of broad and narrow spectrum antibiotics are not consistent across prior cohort studies. While penicillins can be reliably classified as narrow spectrum, other antibiotic classes differ in their targets by generation and we were unable to reliably classify antibiotics by targeted spectrum. Classification of bacteria as having bactericidal (kills bacterial cells) or bacteriostatic (inhibits growth of bacterial cells) action has been used previously in examining associations with diabetes [40, 41], although the distinction has not previously been adopted in studies of associations with obesity or weight gain.

There were several strengths and limitations to this study. Primary limitations include the lack of indications data among non-antibiotic users, the lack of data about shorter-term antibiotic use (e.g., 7–10 days of antibiotics at a time), the entirely female and mostly white makeup of the study population, and the relatively small numbers of long-term users of antibiotics. We were unable to perform a true analysis of the interaction between illness and antibiotics, as we had no data on the relevant illnesses in non-antibiotic users. For instance, if someone reported no antibiotic use, they were not asked about possible antibiotic-related illnesses. Thus, we did not know if non-users had a skin condition or a respiratory condition, and could not evaluate the effect of antibiotics among all participants with a particular condition. Instead, we were limited to estimating associations for participants who used antibiotics for skin purposes, used for non-skin purposes, or did not use that antibiotic. The confidence intervals for most of these associations were quite wide, which limited any assessment of whether the effects were different by indication category. However, we can generally conclude that the associations for penicillins remained consistent regardless of indication, while associations for tetracyclines/bacteriostatics seemed to vary by indication.

Another variable with potential misclassification is self-reported weight, particularly during the participant’s 30’s. Weight may fluctuate significantly over a decade, and it is unknown whether women reported average weight, maximum weight, or minimum weight during their 30s. However, BMI in 30s was similar for antibiotic and non-antibiotics users, suggesting that outcome misclassification is likely non-differential by exposure status. Additionally, we used self-reported weight at both follow-ups, which may be subject to some exposure misclassification, particularly among obese and underweight women. However, comparisons against examiner-measured height and weight in this population suggest that women are accurate self-reporters of their height and weight, and under-reporting for obese women is rarely >10% [42].

Recall bias for the exposure could also be a potential problem in this study. Using an antibiotic three times a week for three months or more is an unusually long period of time for antibiotic usage. Although participants may be more likely to remember taking an antibiotic for this length of time, it is also possible that women who recall taking antibiotics for an unusual length of time, such as one month, may have incorrectly reported taking the antibiotic for the full three months. Thus, the retrospective nature of this medication assessment is subject to some exposure misclassification. Additionally, although long-term antibiotic use may be more likely to impact the microbiome than short-term doses, antibiotic doses for one to two weeks are much more common. Repeated dosings of smaller amounts do appear to bring about long-lasting changes in gut microbiomes [11]. It is possible that many of the women that were classified as unexposed in the current study were actually exposed to multiple dosings of antibiotics during the time periods of interest, but were considered unexposed because they did not meet the criteria of taking antibiotics for 3 months or more at a time.

A final limitation is the external generalizability of the study. We only studied these associations in a female population that was mostly white. Therefore, we cannot draw conclusions about the relevance of these findings to men, or to a population with a higher percentage of minorities. Although we observed minimal confounding, and no effect modification by race/ethnicity, residual confounding may be present. Minority participants in the Sister Study tend to have higher incomes and to have competed more years of schooling than the general population of minority women which could in turn affect factors related to both infection and access to healthcare.

Study strengths include considering indication for use in analyses, using both retrospective and prospective longitudinal data, and examination of this study question in an adult population over a long period of time. The use of both retrospective and prospective data revealed a few notable items. One, there were no associations between antibiotic use in the past 12 months and prospective weight change. It is possible that weight does not change immediately after antibiotic exposure. Changes in satiety hormones and energy storage or harvest may not stabilize until the relevant microbial communities stabilize in response to antibiotic exposure, which may take several years. Second, associations for penicillins were consistent with both retrospective and prospective data.

This potential antibiotic side effect should be further investigated, as public health impact may be high. Antibiotic prescription rates are high; in 2011, healthcare providers prescribed 842 prescriptions per 1000 persons, and antibiotic prescriptions are prescribed incorrectly for between 38% to 60% of ambulatory visits [43, 44]. Perceptions in clinical communities about harmful side effects of antibiotic use are generally limited to concerns about antibiotic resistance, but the possible impact on the microbiota and associated ailments should be considered. Long-term use of antibiotics is somewhat rare, and penicillins are used long-term for prophylactic treatment of rheumatic fever, sickle cell disease, recurrent otitis media, endocarditis, salmonella infections, and certain types of Lyme disease. Although the benefits of antibiotics may outweigh the possible side effects of weight gain in these clinical circumstances, patients should be monitored for weight changes in response to judicious treatment.
